# Thermodynamic and Kinetic Studies of the Precipitation of Double-Doped Amorphous Calcium Phosphate and Its Behaviour in Artificial Saliva

**DOI:** 10.3390/biomimetics9080455

**Published:** 2024-07-25

**Authors:** Kostadinka Sezanova, Rumiana Gergulova, Pavletta Shestakova, Diana Rabadjieva

**Affiliations:** 1 Institute of General and Inorganic Chemistry, Bulgarian Academy of Sciences, Acad. G. Bonchev Str., bl. 11, 1113 Sofia, Bulgaria; ksezanova@abv.bg (K.S.); rumigg@yahoo.com (R.G.); 2 Institute of Organic Chemistry with Centre of Phytochemistry, Bulgarian Academy of Sciences, Acad. G. Bonchev Str., bl. 9, 1113 Sofia, Bulgaria; pavletta.shestakova@orgchm.bas.bg

**Keywords:** double-doped calcium phosphate, artificial saliva, simulated body fluid, thermodynamics, kinetics, solid-state NMR

## Abstract

Simulated body fluid (SBF) and artificial saliva (AS) are used in biomedical and dental research to mimic the physiological conditions of the human body. In this study, the biomimetic precipitation of double-doped amorphous calcium phosphate in SBF and AS are compared by thermodynamic modelling of chemical equilibrium in the SBF/AS-CaCl_2_-MgCl_2_-ZnCl_2_-K_2_HPO_4_-H_2_O and SBF/AS-CaCl_2_-MgCl_2_-ZnCl_2_-K_2_HPO_4_-Glycine/Valine-H_2_O systems. The saturation indices (SIs) of possible precipitate solid phases at pH 6.5, close to pH of AS, pH 7.5, close to pH of SBF, and pH 8.5, chosen by us based on our previous experimental data, were calculated. The results show possible precipitation of the same salts with almost equal SIs in the two biomimetic environments at the studied pHs. A decrease in the saturation indices of magnesium and zinc phosphates in the presence of glycine is a prerequisite for reducing their concentrations in the precipitates. Experimental studies confirmed the thermodynamic predictions. Only X-ray amorphous calcium phosphate with incorporated Mg (5.86–8.85 mol%) and Zn (0.71–2.84 mol%) was obtained in the experimental studies, irrespective of biomimetic media and synthesis route. Solid-state nuclear magnetic resonance (NMR) analysis showed that the synthesis route affects the degree of structural disorder of the precipitates. The lowest concentration of dopant ions was obtained in the presence of glycine. Further, the behaviour of the selected amorphous phase in artificial saliva was studied. The dynamic of Ca^2+^, Mg^2+^, and Zn^2+^ ions between the solid and liquid phases was monitored. Both direct excitation ^31^P NMR spectra and ^1^H-^31^P CP-MAS spectra proved the increase in the nanocrystalline hydroxyapatite phase upon increasing the incubation time in AS, which is more pronounced in samples with lower additives. The effect of the initial concentration of doped ions on the solid phase transformation was assessed by solid-state NMR.

## 1. Introduction

The biomimetic approach in the synthesis and in vitro testing of biomaterials involves the creation of an environment as close as possible to that of the human organism. For bone-like materials, these are the different types of simulated body fluids (SBFs), such as Earle’s balanced salt solution (EBSS) [1] or Hank’s balanced salt solution (HBSS) [2], which contain all major ions of the inorganic part of blood plasma but differ in their Ca/P ratio, the concentration of HCO_3_^−^ ions and pH. A multicomponent inorganic solution with a Ca/P ratio of 2.5, equivalent to blood plasma, known as conventional simulated body fluid (SBFc), was initially prepared by Kokubo [3] and has established itself as the most stable solution over time and, accordingly, the most used in biomedical and dental studies. SBFc differs from blood plasma only in the lower HCO_3_^−^ concentration (4.2 mmol.dm^−3^ versus 27 mmol.dm^−3^). Bayractar and Tas [4] revised the SBFc by increasing the HCO_3_^−^ concentration to 27 mmol.dm^−3^, but this solution is unstable, decreasing its HCO_3_^−^ concentration in open-air experiments. Considering materials for tooth restoration and dental disease prevention, artificial saliva (AS) is an optimal choice. Natural saliva is a complex system with many components and variables depending on the individual, diet, and health. Except that the composition of saliva is even affected by what time of day it is collected [5]. Similarly to SBFs, natural saliva consists of inorganic electrolytes, such as Na^+^, K^+^, Mg^2+^, Ca^2+^, and H_n_PO_4_^3−n^ ions, low-weight organic substances, such as urea and uric acid, as well as high-weight organic substances, such as immunoglobulins, proteins, enzymes, and mucins [5,6,7]. Nunes et al. [7] compared the composition of saliva and blood plasma, revealing a higher content of organic compounds and a lower content of electrolytes in saliva than in blood plasma. Despite their low content, inorganic ions maintain saliva’s pH balance and osmotic pressure and provide a natural remineralisation medium against early carious lesions. The precipitation of calcium phosphates in simulated body fluids to produce bone-like biomaterials has been the subject of intensive research [8,9,10,11,12], while this process in an artificial saliva medium is rather incidental [13,14,15].

In biomedical engineering, metal-doped calcium phosphate biomaterials are a promising class of materials with specific properties that meet the requirements of tissue engineering, drug delivery systems, regenerative medicine, etc. In recent years, much work has been performed on doping calcium phosphates with transition metals like Fe, Co, Cu, Mn, etc. Ribeiro et al. [16] have produced and characterised iron-doped calcium phosphate nanoparticles as a fully biodegradable substitute for the current chemodynamic drugs. Incorporated iron with different oxidation states provided the nanoparticles with varying magnetic properties, leading to a higher magnetic resonance imaging contrast. Biphasic calcium phosphate ceramics doped with cobalt showed antibacterial activity [17]. They also enhanced angiogenic marker expression and elevated cell viability compared to pure ceramics. Antibacterial activity also possesses copper-doped ceramics [18] and bio-glasses [19]. A mesoporous bioactive glass containing copper has been shown to stimulate angiogenesis in an in vivo zebrafish model [20]. Despite the improved properties, the application of biomaterials containing transition metals is limited by their concentrations. Some can exhibit toxic effects and lowered biocompatibility on biological systems, especially at higher concentrations.

Zinc and magnesium are essential elements that play crucial roles in the human body. The balance of these minerals in hard tissues is critical for maintaining bone and teeth health. Zinc is known to play a role in bone metabolism. Its concentration in hard tissue ranges from 60 to 200 μg/g [21,22]. Magnesium contributes to bone structure and density. Its concentration is several times higher than Zn’s and varies from approximately 300 to 600 μg/g [23]. These concentrations depend on the individual’s bone type, age, and health status. Magnesium and zinc are also essential for dental and oral health. They improve the antimicrobial microenvironment, reduce inflammatory processes in the oral cavity, and are considered to improve the flexibility of tooth enamel [24]. Additionally, because calcium phosphate-based remineralisation systems can release Ca^2+^ and PO_4_^3−^ ions, increasing salivary supersaturation concerning the hydroxyapatite phase, they are appropriate for enamel remineralisation [25,26,27].

This work aims to compare the effects of the electrolyte environment of simulated body fluid and artificial saliva and the inclusion of some organic molecules on the chemical and phase composition of biomimetic precipitated calcium phosphates double-doped with Mg and Zn. Glycine (Gly) and valine (Val) were chosen as organic additives because amino acid residues are involved in the structure of non-collagen proteins and have also been found to be present in blood plasma [28]. Two complementary approaches are applied in our research: a thermodynamic approach for predicting the behaviour of the systems and different experimental synthesis routes. Saturated indices of possible solid phases in the SBF/AS-NaCl-CaCl_2_-MgCl_2_-ZnCl_2_-K_2_HPO_4_-H_2_O and SBF/AS-NaCl-CaCl_2_-MgCl_2_-ZnCl_2_-K_2_HPO_4_-Gly/Val-H_2_O systems were calculated by applying the thermodynamic equilibrium model and the influence of the chemical composition of the starting solutions on the composition of the resulting precipitates was evaluated. The results of the thermodynamic prognosis were proved experimentally. Furthermore, the release of Ca^2+^, Mg^2+^, and Zn^2+^ ions from selected samples into an artificial saliva solution was analysed to assess the potential for promoting enamel remineralisation.

## 2. Materials and Methods

### 2.1. Solution Used

This study used modified simulated body fluids (mSBFs) and modified artificial salivas (mASs) (Table 1).

The mSBFs were created based on a conventional simulated body fluid (SBFc) [3], while mASs were based on composition, as proposed by Klimek et al. [29]. The solutions were prepared by mixing the corresponding chemical substances to obtain the concentrations in Table 1. The following substances were used: NaCl (INEOS, A.R., London, UK), NaHCO_3_ (SOLVAY, A.R., Brussels, Belgium), K_2_HPO_4_.3H_2_O (Merck, A.R., Darmstadt, Germany), MgCl_2_.6H_2_O, (Merck, Darmstadt, Germany), CaCl_2_.2H_2_O, (Sigma-Aldrich, A.R., St. Louis, MO, USA), Na_2_SO_4_ (JLC-CHEMIE Hendel GmbH, A.R., Wohlen bei Bern, Switzerland), KH_2_PO_4_ (Merck, Darmstatd, Germany), Na_2_HPO_4_ (Merck, Darmstatd, Germany), KCl, (INEOS, A.R., London, UK), NaSCN (Sigma-Aldrich, St. Louis, MO, USA, ACS reagent, ≥98.0%), and NH4Cl (Reanal, Budapest, Hungary).

The solutions mSBFc1 and mAS1 were free of M^2+^ (M^2+^ = Ca, Mg, Zn), but mSBFc2, mSBFc3, mAS2, and mAS3 were phosphorous- and carbonate-free. Thus, preliminary precipitation was avoided. The concentrations of Gly and Val were chosen to be as close as possible to their solubility at 25 °C but not to cause solid phase crystallisation at room temperature (18–28 °C).

The pH of mSBFc1, mSBFc2, mSA1, and mSA2 was adjusted to 8.0–8.2 using 0.05 M tris (hydroxymethyl) aminomethane (SIGMA, A.R.) for modified SBF solutions and 1 M KOH for modified AS solutions. Zinc solutions (mSBFc3 and mAS3) were prepared separately to prevent hydrolysis, and their pHs remained unchanged.

Concentrations of Mg^2+^, Zn^2+^, Ca^2+^, and PO_4_^3−^ ions were calculated to produce calcium phosphate precursors with ratios Mg^2+^/(Mg^2+^ + Zn^2+^ + Ca^2+^) = 7 mol%, Zn^2+^/(Mg^2+^ + Zn^2+^ + Ca^2+^) = 3 mol%, and (Mg^2+^ + Zn^2+^ + Ca^2+^)/P = 1.67, Then, the concentration of Mg^2+^ was doubled to provide the desired percentage of magnesium ions in the structure of the precipitates [30]. The concentrations of Mg^2+^ and Zn^2+^ ions were selected based on our previous studies on the biocompatibility of Mg- or Zn-modified tri-calcium phosphates [31], and as well as on the presumption that the concentration of Zn^2+^ ions is lower than that of Mg^2+^ in the hard tissues.

### 2.2. Biomimetic Precipitation

Three sets of experiments were carried out, depending on the mixing sequence of the starting solutions. The experimental conditions are presented in Table 2.

All experiments were carried out with the same volumes of each initial solution at room temperature, intensive steering, and pH 8–8.2 keeping it with 1 mol.dm^−1^ KOH. The solutions were added at a 3 mL/min rate in Series B and C. The suspensions were left in the mother liquor for one hour while constantly stirring. They were then washed with water several times through decantation and freeze-dried.

### 2.3. In Vitro Test in Artificial Saliva

Freeze-dried precipitates of CP-SBFgb or CP-SBFgbGly (0.25 g of each) were placed in 15 mL of artificial saliva solutions (ASS) with a composition according to Klimek [29] (Table 1) under static conditions in closed plastic vessels. In addition to the inorganic components described in Section 2.1, the AS solution also contains urea (Sigma-Aldrich, A.R., St. Louis, MO, USA), glucose (Sigma-Aldrich, A.R., St. Louis, MO, USA), ascorbic acid (Sigma-Aldrich, A.R., St. Louis, MO, USA), and mucin from porcine stomach type II (Sigma-Aldrich, St. Louis, MO, USA) in concentration shown in Table 1. The pH was 6.3. The commercial product mucin was purified using the dissolution/lyophilisation process.

The solid samples were kept in the artificial saliva solutions for 1, 2, 4, 6, 24, 48, 72, 240, 480, and 720 h, respectively, at 37° ± 1 °C. The solutions were regularly replaced twice weekly, starting from the 3rd day (72nd h). At the appropriate time, the sample was filtered on a blue strip using a vacuum pump, and the solid residue was washed three times with 15 mL of distilled water and then dried at 60 °C for 24 h.

### 2.4. Characterizations

#### 2.4.1. Chemical Analysis

The sum of Ca^2+^, Zn^2+^, and Mg^2+^ ions in the solid and liquid samples was determined complexometrically with EDTA at pH 10 and indicator Eriochrom Black T. The concentrations of Zn^2+^ and Mg^2+^ ions were determined by ICP-OES (PRODIGY 7, Teledyne, Leeman Labs, Hudson, NH, USA). Spectrophotometer NOVA 60 (Merck KGaA, Darmstadt, Germany) and Merck Spectroquant test kits (Merck KGaA, Darmstadt, Germany) were used to determine the concentrations of PO_4_^3−^ ions. The solids were dissolved in nitric acid.

Ten parallel independent measurements were performed for each assay. The statistical analysis of the data was carried out using Microsoft Excel 2016. The standard deviation value expresses the accuracy of the results. The accuracy of the (Ca + Mg + Zn)/P ratio was calculated using Equation (1) and M/(Ca + Mg + Zn) (M = Mg, Zn), using Equation (2):

(1) $$SD=\sqrt{\frac{s_{\left(Ca+Zn+Mg\right)}^{2}}{C_{\left(Ca+Zn+Mg\right)}}}+\sqrt{\frac{s_{P}^{2}}{C_{P}}}$$

(2) $$SD,\;\%=\left(\sqrt{\frac{s_{M}^{2}}{C_{M}}}+\sqrt{\frac{s_{\left(Ca+Zn+Mg\right)}^{2}}{C_{\left(Ca+Zn+Mg\right)}}}\right)\;*\;100$$

where *SD*—standard deviation; *S_(Ca+Mg+Zn)_*, *S_P_*_,_ and *S_M_*—standard deviation of measurements of sum of Ca^2+^, Zn^2+^, and Mg^2+^ ions, P (PO_4_^3−^ ions), and M (Zn^2+^ and Mg^2+^), respectively; *C_(Ca+Mg+Zn)_, C_P,_* and *C_M_*—average value of measurements of concentration of sum of Ca^2+^, Zn^2+^, and Mg^2+^ ions, P(PO_4_^3−^ ions), and M (Zn^2+^ and Mg^2+^), respectively.

#### 2.4.2. X-ray Diffraction Analysis (XRD)

A Bruker D8 Advance diffractometer (Bruker AXS Advanced X-ray Solutions GmbH, Karlsruhe, Germany) was used to perform powder XRD. The X-ray source was a Cu tube (λ = 1.5418 Å). The pattern record was made using a LynxEye detector (Bruker AXS GmbH, Karlsruhe, Germany). The data were gathered in the 10 to 90° 2θ range with a step of 0.03° 2θ and a counting rate of 57 s/step for the phase identification. With the aid of the ICDD-PDF2 (2014) database and Diffracplus EVA software (v. 4, 2014), the phase composition was determined.

#### 2.4.3. Solid-State Nuclear Magnetic Resonance (NMR) Analysis

NMR spectra were recorded on a Bruker Avance III 600 spectrometer(Karlsruhe, Germany), ^1^H working frequency 599.98 MHz, 242.84 MHz for ^31^P, using 4 mm ^1^H/^31^P-^15^N solid-state iProbe CP-MAS dual ^1^H/X probe head (Bruker BioSpin GmbH, Karlsruhe, Germany). The samples were loaded in 4 mm zirconia rotors and spun at a magic angle spinning (MAS) rate of 10 kHz for ^31^P and at 14 kHz for ^1^H measurements. The quantitative direct excitation ^31^P NMR spectra were recorded with a “single pulse” sequence (Bruker Topspin library), 90° pulse length of 3.1 μs, 8 K time domain data points, spectrum width of 73.5 kHz, 256 scans, and a relaxation delay of 150 s. The spectra were processed with an exponential window function (line broadening factor 5) and zero filled to 32 K data points. The ^1^H → ^31^P cross-polarisation MAS (CP-MAS) spectra were acquired with the following experimental parameters: ^1^H excitation pulse of 3.4 μs, 10 s relaxation delay, 256 scans were accumulated, and the MAS rate was 10 kHz. Series of spectra with contact time varied from 200 µs up to 6 ms were measured. ^1^H SPINAL-64 decoupling scheme was used during the acquisition of CP experiments. All ^31^P chemical shifts were referenced against the external solid reference NH_4_H_2_PO_4_ (δ 0.9 ppm). The DMfit software (dmfit 1.0) was used for the deconvolution, simulation, and fitting of the experimental NMR data [33].

#### 2.4.4. Thermodynamic Modelling

Thermodynamic modelling of chemical equilibrium in the SBF/AS-CaCl_2_-MgCl_2_-ZnCl_2_-K_2_HPO_4_-H_2_O and SBF/AS-CaCl_2_-MgCl_2_-ZnCl_2_-K_2_HPO_4_-Gly/Va-H_2_O systems were performed using the PHREEQCI v.3.3.7.11094 computer program [34].

The calculation of possible precipitation was performed by simulating the mixing of equal volumes of the modified solutions, mSBFc2, mSBFc3, mSBFc1 or mAS2, mAS3, and mSAS1 (Table 1), at a pH of 6.5, close to the pH of the saliva, 7.5, close to the pH of the blood plasma, and 8.5, chosen by us based on our previous experimental data, and a temperature of 25 °C.

The saturation indices (SIs) used as an indicator for possible salt precipitation were calculated according to Equation (3):

SI = lg(IAP/K)(3)

where IAP is the ion activity product, and K is the solubility product.

Thermodynamic calculation of SI at in vitro test with AS was performed using the computer program VisualMinteq v.3 (https://vminteq.com/, accessed on 5 December 2021), where the sum of concentrations of organic substances in AS (urea, ascorbic acid, glucose, and mucin) was entered as a total concentration of dissolved organic matter.

Both computer codes are based on the ion-association model. A mass-action expression with appropriate solubility product constants defined salt precipitations. The activity coefficients of all possible simple and complex species were calculated using the extended Debye–Hückel theory.

When SI > 0, the solution is supersaturated concerning a particular salt, and it will precipitate; when SI < 0, the solution is undersaturated, and the salt will not precipitate; when SI = 0, the solution and the salt will be in equilibrium.

The compositions of the SBFs and ASs used as input data in the calculations are provided in Table 1. Only reactions of association/dissociation and dissolution/crystallisation between the cations and anions were considered. We used an expanded database with all the necessary thermodynamic formation constants [35,36].

High-temperature phases, α- and β-Ca_3_(PO_4_)_2_, Mg_3_(PO_4_)_2,_ and Ca_4_(PO_4_)_2_O, phases resulting from dehydration as CaHPO_4_ and long-term maturated phases CaMg(CO_3_)_2_, CaMg_3_(CO_3_)_4_, Zn_2_(OH)_3_Cl, Zn_4_(OH)_6_SO_4_, Zn_5_(OH)_8_Cl_2_, and phases with a negative SI were excluded from the model. Ca_9_Mg(HPO_4_)(PO_4_)_6_ was used as an example of Mg-doped calcium phosphate for which the thermodynamic precipitation constant is known. Double-doped calcium phosphates, or this one doped with Zn, were not included in the model because of a lack of data.

## 3. Results

### 3.1. Thermodynamic Modelling of the Precipitation Process

**Figure 1:**
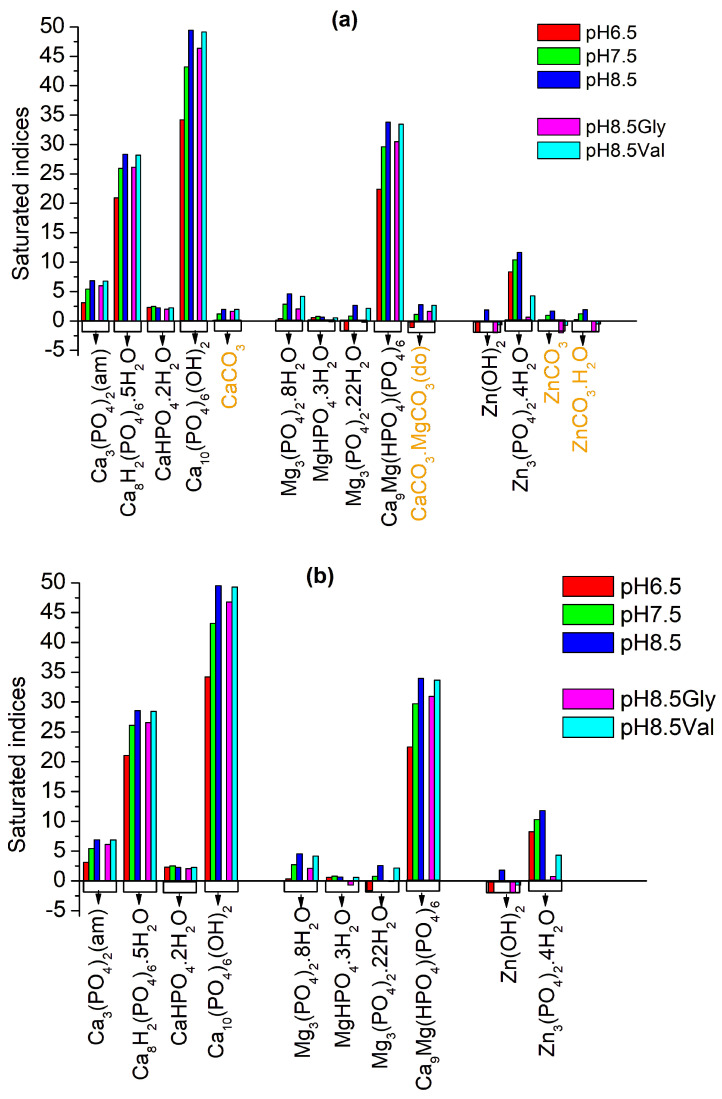
Calculated saturation indices (SIs) of the possible precipitated salts in the biomimetic systems with the participation of SBF (**a**) and AS (**b**) at pH 6.5, 7.5, and 8.5. (am) denotes amorphous phase; (do) denotes disordered phase; Gly denotes participation of glycine in the initial solutions; and Val denotes participation of valine in the initial solutions.

Calculated saturation indices (SIs) of the salts that might have precipitated in the biomimetic SBF/AS-CaCl_2_-MgCl_2_-ZnCl_2_-K_2_HPO_4_-H_2_O and SBF/AS-CaCl_2_-MgCl_2_-ZnCl_2_-K_2_HPO_4_-Gly/Val-H_2_O systems under study are presented in Figure 1.

The results show similar behaviour of the systems containing SBF and AS concerning the phosphate salts that can precipitate (SI > 0) regardless of the pH of the medium and the presence of glycine or valine. The difference is in the possible co-crystallisation of carbonate salts in the systems with the participation of SBF but without including glycine and valine. This is also the behaviour of Zn(OH)_2_, although it also appears in the systems with the participation of AS. Precipitation of all four calcium phosphate salts, CaHPO_4_.2H_2_O, Ca_3_(PO_4_)_2_(am)_,_ Ca_8_H_2_(PO_4_)_6_.5H_2_O, and Ca_10_(PO_4_)_6_(OH)_2,_ is thermodynamically possible at the three pHs, 6.5, 7.5, and 8.5, and only for CaHPO_4_.2H_2_O. This possibility decreases with increasing pH (SI values decrease).

As expected, Ca_10_(PO_4_)_6_(OH)_2_ is the most thermodynamically stable. Of the magnesium phosphate salts, the most thermodynamically stable is Ca_9_Mg(HPO_4_)(PO_4_)_6_, whose SIs are second only to those of Ca_10_(PO_4_)_6_(OH)_2_., i.e., the preparation of divalent ion-doped calcium phosphates is thermodynamically advantageous. Mg_3_(PO_4_)_2_.22H_2_O cannot precipitate at pH 6.5, and MgHPO_4_.3H_2_O cannot precipitate in the syntheses involving glycine. Zn_3_(PO_4_)_2_.4H_2_O is possible to precipitate in all cases. In the presence of Gly and Val, SIs of the potential to precipitate salts decrease. When Val is included, SIs of calcium and magnesium salts are reduced insignificantly, while at Zn_3_(PO_4_)_2_.4H_2_O reaches a 55% reduction. In the presence of Gly, the reduction is between 2 and 5% by calcium phosphate and between 50 and 100% by magnesium and zinc salts.

### 3.2. Experimental Studies on the Biomimetic Synthesis

The experimental studies were carried out at pH 8–8.2 since thermodynamic calculations showed similar results at the three pHs: 6.5, close to the saliva, 7.5, close to the blood plasma, and 8.5, chosen by us based on previous experimental data on precipitation of calcium phosphates [35,36]. The pH range was chosen to avoid precipitation of acidic calcium phosphate salts, which are not characteristic of hard tissues.

**Figure 2:**
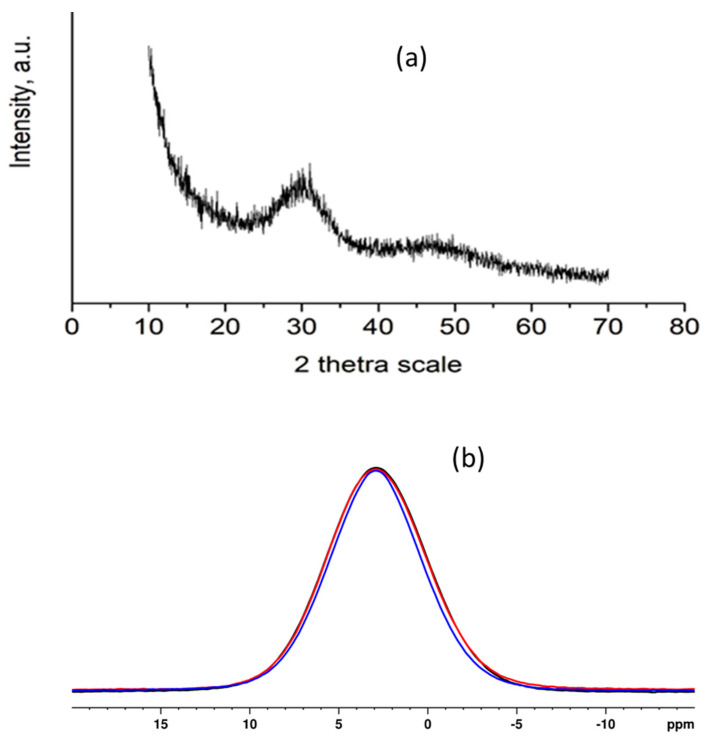
X-ray powder pattern of the sample obtained in Series B in the presence of AS and valine (**a**,**b**) direct excitation ^31^P NMR spectra of CP-AScp (black), CP-ASf (red), and CP-ASgb (blue) samples.

Experimental studies showed that X-ray amorphous calcium phosphate (Figure 2) with (Ca + Zn + Mg)/P molar ratio lower than that of hydroxyapatite (Table 3) was always obtained regardless of the electrolyte environment or the participation of glycine and valine. Since all X-ray powder patterns are the same (see Figure S1 in Supplementary Materials), the X-ray pattern of only a sample of Series B is shown as an example—a sample precipitated in the presence of artificial saliva and valine (Figure 2a).

Solid-state NMR spectroscopy was applied to gain a better insight into the structural details at the molecular level of the samples obtained by the three methods. As an example, direct excitation ^31^P NMR spectra of CP-ASf (Series A), CP-AScp (Series B), and CP-ASgb (Series C) samples are provided in Figure 2b. The spectra of the three samples show one broad resonance centred at 1.90 ppm, indicating the formation of a disordered amorphous apatite phase. The linewidths of the signals of CP-ASf (Series A) and CP-AScp (Series B) samples are slightly broader (1600 Hz) as compared to the linewidth of the resonance of CP-ASgb (Series C) sample (1500 Hz). Therefore, we suggest that CP-ASgb (Series C) material is characterised by an overall lower degree of structural disorder.

The inclusion of Mg^2+^ and Zn^2+^ ions in the solid phase varies from 5.86 to 8.85 mol% for Mg^2+^ and between 0.71 and 2.84 mol% for Zn^2+^ jons (Table 3).

The chemical composition of the samples in the different series (A, B, and C) obtained in the two electrolyte media, SBF or AS, is close. The concentrations of Mg and Zn vary within very narrow limits within one series. In the pure electrolyte systems (without the participation of Gly and Val), the lowest Mg concentrations were found in the precipitates of Series A, followed by those of Series C and B. For Zn, the lowest values were obtained in the solid phases of Series B, and the concentrations of Zn in Series A and C are close. In the same method of obtaining (Series C) and equal pH 8–8.2, but with the participation of Gly and Val, the amount of dopant ions decreases, and this decrease is most significant for CP-SBFgbGly and CP-ASgbGly.

### 3.3. In Vitro Test in Artificial Saliva

**Figure 3:**
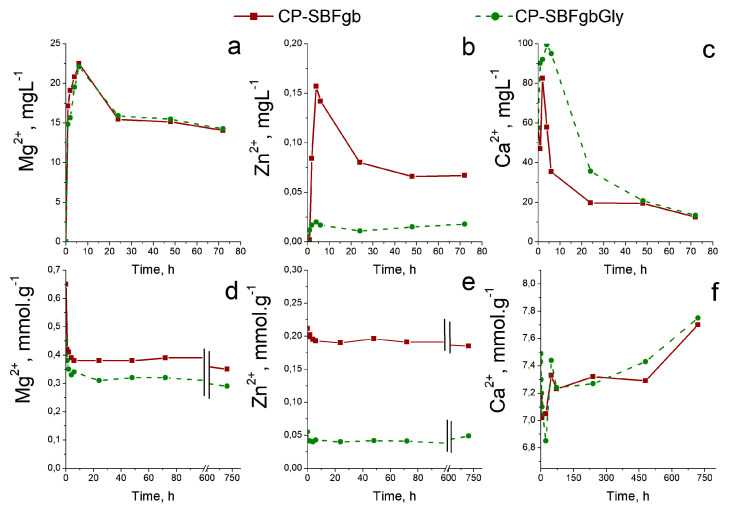
Kinetic curves of Mg^2+^, Zn^2+^, and Ca^2+^ contents in liquid (**a**–**c**) and solid (**d**–**f**) phases after different contact times in AS.

To study the behaviour of the obtained precipitates in an artificial saliva environment, we chose CP-SBFgbGly and CP-SBFgb due to the most significant difference in the concentrations of Mg and Zn in the precipitated solid phases (Table 3). We monitored the changes in the contents of Mg^2+^, Zn^2+^, and Ca^2+^ ions in the liquid (up to the 72nd h, before the replacement of the artificial saliva) and solid phases (up to the 720th h) with time. The results showed a similar behaviour of the two studied solid phases, manifested by changes in the composition of both solid and liquid phases (Figure 3), most clearly expressed during the first 24 h.

The concentrations of all three investigated ions in the liquid phase increased, reaching a maximum between the 2nd and 6th h, after which they decreased. After the 24th to the 72nd h, they remained practically unchanged for Mg^2+^ and Zn^2+^ ions and continued to decline for Ca^2+^ ions. Changes in the solid phase corresponded to those in the liquid. After the 72nd h, an additional slight decrease in the content of Mg^2+^ and Zn^2+^ ions and an increase in the content of Ca^2+^ ions were observed in the solid phases.

The concentrations of Mg^2+^ ions in the liquid and solid phases were very close for the two studied samples. A difference was observed in the concentrations of Ca^2+^ and Zn^2+^ ions, the largest in the case of Zn^2+^ ions, due to the more significant difference in their starting concentrations (Table 3).

Selected samples were characterised by solid-state NMR spectroscopy using direct excitation ^31^P and ^1^H-^31^P cross-polarisation magic angle spinning (CP-MAS) techniques. The direct excitation ^31^P spectra provide quantitative information about the relative fractions of the different calcium phosphate phases, while the ^1^H → ^31^P CP-MAS spectra allow for qualitatively identifying the presence of acidic hydrogen phosphate species.

**Figure 4:**
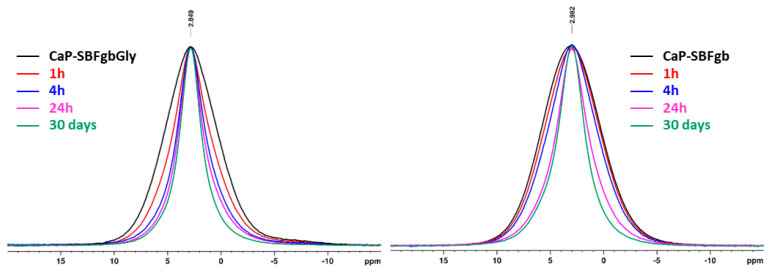
Direct excitation ^31^P NMR spectra of CaP-SBFgbGly and CaP-SBFgb samples incubated at different periods in AS: parent samples (black), 1 h in AS (red), 4 h in AS (blue), 24 h in AS (magenta), and 30 days in AS (green).

Figure 4 presents the direct excitation ^31^P spectra of the samples from the two series. The spectra of the parent CaP-SBFgbGly and CaP-SBFgb represent a broad spectral pattern centred at around 2.9 ppm that is characteristic of the amorphous apatite phase. The overall linewidth of the resonance of the CaP-SBFgb was slightly broader (1562 Hz) compared to the CaP-SBFgbGly (1434 Hz).

**Figure 5:**
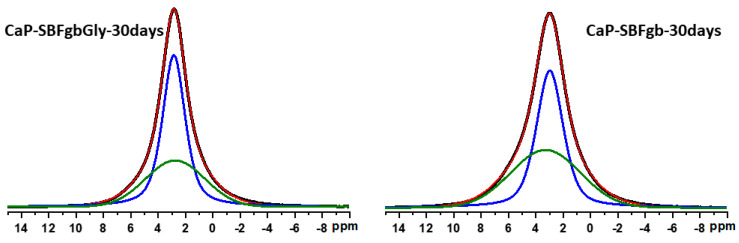
Direct excitation ^31^P NMR spectra of the samples CaP-SBFgbGly-30 days and CaP-SBFgb-30 days incubated in AS for 30 days. The experimental spectra are in black, while the simulated spectra are in red lines. The deconvoluted ^31^P spectra show the individual contributions of the two components, indicating the presence of crystalline (blue) and disordered amorphous phase (green).

Within each series, a significant signal narrowing was observed with increased incubation time in the AS solution. The narrowing of the signals indicates the presence of both an amorphous apatite phase (broader resonance) and a nanocrystalline phase of hydroxyapatite (more narrow resonance) [37,38]. The discrimination of the two phases from the ^31^P spectra is not straightforward due to their almost identical chemical shifts and the overlap of their resonance. A closer look at the behaviours of the two series as a function of the incubation time in AS solution showed that in the case of the CaP-SBFgb series, the linewidth of the resonance remained similar to the linewidth of the parent CaP-SBFgb sample until 4 h of incubation. At the same time, a significant signal narrowing was observed after 24 h of incubation in AS. The ^31^Pspectra of CaP-SBFgbGly samples obtained at different incubation times showed that the crystallisation process started much earlier, and already at 1 h of incubation, there is a relatively high amount of nanocrystalline phase as indicated by the significant signal narrowing. The deconvolution of the spectral pattern of the samples after 30 days of incubation in AS solution allowed quantification of the amount of the amorphous and crystalline phases in the two series (Figure 5). In the CaP-SBFgbGly-30 days sample, the ratio of crystalline to amorphous phase was 61:39, while in the CaP-SBFgb-30 days sample, the amount of crystalline phase was 52%, and a higher amount of amorphous phase was determined (48%).

**Figure 6:**
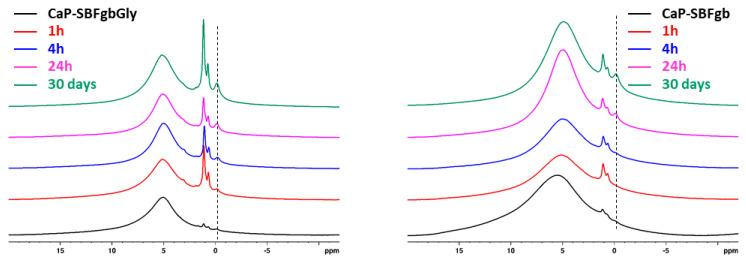
^1^H NMR spectra of CaP-SBFgbGly and CaP-SBFgb samples incubated at different periods in AS: parent samples (black), 1 h in AS (red), 4 h in AS (blue), 24 h in AS (magenta), and 30 days in AS (green). The vertical dotted line indicates the increase in the characteristic resonance of the nanocrystalline HAp phase with increased incubation time.

The increase in the amount of nanocrystalline hydroxyapatite phase upon increasing the incubation time in AS is further evidenced by the systematic increase in the intensity of the signal at around −0.5 ppm in the ^1^H spectra, which is characteristic of hydroxyapatite (Figure 6) [38,39]. The ^1^H spectra also show a strong water peak at around 4.5–5.5 ppm originating from the bulk water, as well as several weak and narrow resonances between 0 and 1 ppm that could be assigned to the mobile water molecules at the surface of the particles. The broad low-intensity resonances in ^1^H spectra between 10 and 15 ppm indicated the presence of a small amount of some acidic P-OH moieties, like the HPO_4_^2−^ group. This resonance is better visible in the spin-echo experiment, where the broad water resonance at around 5 ppm is partially filtered out (Figure S2 in Supplementary Materials).

**Figure 7:**
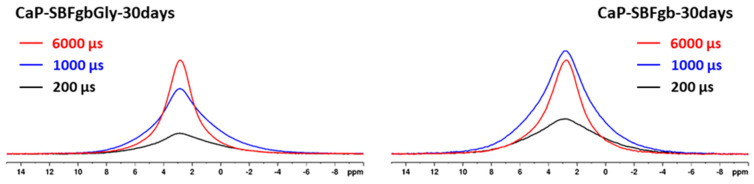
^1^H-^31^P CP-MAS spectra of the samples CaP-SBFgbGly-30 days and CaP-SBFgb-30 days at three different mixing times of 200 µs (black), 1000 µs (blue), and 6000 µs (red).

The simultaneous presence of crystalline and amorphous phases was also evidenced by ^1^H → ^31^P CP-MAS spectra. In this technique, the resonances of ^31^P sites in close proximity to protons are selectively enhanced due to the transfer of magnetisation from the neighbouring protons to the phosphorus nuclei. The effectiveness of cross-polarisation transfer depends on various factors, including ^31^P---^1^H internuclear distance, the number of neighbouring ^1^H, the relaxation rates, and the local dynamics of the structural fragments. These parameters vary from one chemical environment to another, therefore making the CP-MAS technique generally not quantitative. Figure 7 shows as an example the ^1^H → ^31^P CP spectra of the parent CaP-SBFgbGly and CaP-SBFgb samples as well as of CaP-SBFgbGly-30 days and CaP-SBFgb-30 days at three different mixing times of 200 µs, 1000 µs and 3000 µs. At short mixing times, the broad resonance component is predominantly enhanced in the ^1^H → ^31^P CP spectra, while at longer mixing times, the intensity of the narrow signal component increases. These observations could be explained by the specific structural and compositional characteristics of the two phases. The disordered amorphous phase containing hydrogen phosphate protons and water molecules is therefore characterised by a generally more considerable total amount of protons, resulting in efficient ^1^H → ^31^P magnetisation transfer even at short mixing times. In the nanocrystalline hydroxyapatite, however, the OH protons are not part of the phosphate group; the distance to the neighbouring protons is much more significant, and subsequently, longer mixing times are needed for sufficient signal enhancement [38,39].

## 4. Discussion

Calcium orthophosphates are biocompatible compounds with a chemical composition close to hard tissues, making them suitable for application in orthopaedics and dentistry. They are sparingly soluble in water, and their preparation through precipitation is technically feasible and economically viable. However, obtaining monophasic samples is hampered by the need to observe strictly controlled synthesis conditions. The task is further complicated by the inclusion in their composition of modifying ions that extend their key physiologically significant characteristics.

This paper compares the precipitation of Zn- and Mg-doped calcium phosphates in two modified biomimetic media: SBF and AS. The latter share some common inorganic ions to mimic the inorganic composition, ionic strength, and pH of natural fluids. Still, they differ in specific ion concentrations that may affect the phase and chemical composition of the precipitates.

The thermodynamic approach used by us to model the chemical equilibria in the SBF/AS-CaCl_2_-MgCl_2_-ZnCl_2_-K_2_HPO_4_-H_2_O systems allowed us to predict the precipitation processes at three different pHs: 6.5, close to the pH of saliva, 7.5, close to the pH of blood plasma, and 8.5 chosen by us based on our previous experimental data. The results (Figure 1) showed that in both modified biomimetic media, SBF and AS, at all three pHs, it is possible to precipitate the same phosphate salts with almost equal SI, with the sequence of SI reduction as follows: Ca_10_(PO_4_)_6_(OH)_2_ > Ca_9_Mg(HPO_4_)(PO_4_)_6_ > Ca_8_H_2_(PO_4_)_6_.5H_2_O > Zn_3_(PO_4_)_2_.4H_2_O > Ca_3_(PO_4_)_2_(am) > Mg_3_(PO_4_)_2_.8H_2_O > CaHPO_4_.2H_2_O > Mg_3_(PO_4_)_2_.22H_2_O > Mg_3_(PO_4_)_2_.3H_2_O. Carbonate salts were calculated to co-precipitate in the SBF solutions due to the presence of carbonate ions in their compositions. This gives us reason to predict that at a controlled pH ranging from 6.5 to 8.5, the same calcium phosphates will precipitate in both modified biomimetic media. Bearing in mind literary sources on precipitation in SBF [8,40] and our previous studies in an SBF environment [35,36,41], we can assume that in an AS environment, not thermodynamic but kinetic reasons will again be the leading factors in the precipitation of calcium phosphates and at pH 8–8.5 will precipitate not hydroxyapatite but amorphous calcium phosphate. Almost the same saturation indices were calculated for all phosphate salts at pH 8.5 in the systems without the participation of organic molecules and with the involvement of Val. In the syntheses involving Gly, SIs decrease, with the most significant values in Zn_3_(PO_4_)_2_.4H_2_O, followed by magnesium phosphate and calcium phosphate salts. One can expect a difference in the inclusions of the dopant ions in the systems without and with the participation of organic additives, which are more pronounced in the presence of Gly.

The experimental studies were designed at pH 8–8.2 to minimise the possibility of nucleation of acid calcium phosphate salts. The experiments confirmed our prognosis and X-ray amorphous calcium phosphate was obtained in all studied systems. The result is expected because faster kinetics of crystallisation are shown by those salts whose crystal structures are built from chemical entities existing in the solution [42]. At pH 8–8.2, Posner’s clusters form in the calcium phosphate solutions, which build up the structure of amorphous calcium phosphate [43]. On the other hand, Mg^2+^ and Zn^2+^ ions in the solution slow down the crystallisation of hydroxyapatite and stimulate the formation of amorphous calcium phosphate [44]. The similar chemical characteristics and ionic radii of Mg^2+^, Zn^2+^, and Ca^2+^ ions facilitate the substitution of Ca^2+^ ions by Zn and Mg and the formation of substituted calcium phosphates.

The inorganic electrolyte environment in SBF and AS does not affect the degree of incorporation of Mg^2+^ and Zn^2+^ ions into the precipitates (Table 3). The tendency established in our previous studies [30,35] for partially including the initial Mg^2+^ ions in the precipitate is preserved in both media. The reason is the need to overcome the energy barrier for dehydration of Mg(H_2_O)_6_^2+^ complexes stable in the solution. Fast mixing of the solutions (Series A) provides the shortest synthesis time and the lowest concentration of Mg in the solid phase. Continuous precipitation (Series B and C) provides the same synthesis time, but the amount of HPO_4_^2−^ ions surrounding the cations differs. They are dominant in syntheses of Series B, leading to the formation of Zn–phosphate complexes, which are more stable than those of Mg. The concentration of Zn in the solid phases is lower, but that of Mg is the highest compared to Series A and C. Based on the NMR analysis (Figure 2b), which shows a lower degree of structural disorder in the sample of Series C as well as on our long-term research that the precipitation conditions in the C series are the most stable in terms of dopant ion concentrations over repeated experiments, we consider this precipitation method the most suitable for precipitation of calcium phosphates. Dropping all solutions simultaneously ensures a constant microenvironment during precipitation, and the glycine buffer maintains a constant pH throughout the experimental volume. Therefore, we performed precipitation in the presence of Gly and Val using continuous precipitation under glycine buffer. Gly and Val can form stable complexes with the divalent ions in the solution through their carboxyl groups, which inhibit the incorporation of Zn and Mg into the calcium phosphate structure. The stability of Gly complexes is higher than that of Val, and the stability of Zn-Gly/Val complexes is higher than that of magnesium [36]. This determines the lower concentrations of Mg and Zn in the precipitates, which are the lowest in the presence of Gly (Table 3).

Amorphous calcium phosphate is a precursor for hydroxyapatite formation because it is the first compound precipitating from calcium phosphate solutions in alkaline conditions [45,46]. Its application is limited in orthopaedics due to its high reactivity, which causes a higher dissolution rate than new bone deposition. This property would be an advantage in dentistry for enamel restoration of early carious lesions. To verify the possibility of incorporating our materials into remineralisation systems, we conducted preliminary studies to evaluate their behaviour in an artificial saliva environment. The selected samples were obtained by the same method (Series C) and differed maximally in the concentrations of Zn and Mg. The results show that in the first 6 h, the amorphous calcium phosphate dissolves, accompanied by an increase in the concentration of divalent ions in the solution and a decrease in the solid phase. Dissolution leads to an increase in supersaturation concerning calcium phosphates, the critical value of which is reached in the second hour at sample CP-SBFgbGly and the 6th h at CP-SBFgb (Figure 3c). A process of reverse deposition of a solid phase begins, accompanied by recrystallisation of the amorphous product and its transformation into a nanosized crystalline apatite phase (Figure 4, Figure 5, Figure 6 and Figure 7). Solid-state NMR spectra reveal that the concentration of dopants affects the rate of the solid phase transformation. The higher Zn and Mg content in the starting amorphous CP-SBFgb than in CP-SBFgbGly delays the appearance of nanocrystalline hydroxyapatite by about three h (Figure 4). This delay continues, and even after 30 days of contact with AS, the part of the amorphous phase in CP-SBFgb is more significant than in CP-SBFgbGly (Figure 5, Figure 6 and Figure 7). Moreover, NMR spectra show that 30 days is insufficient for a complete phase transformation into a nanocrystalline product.

**Figure 8:**
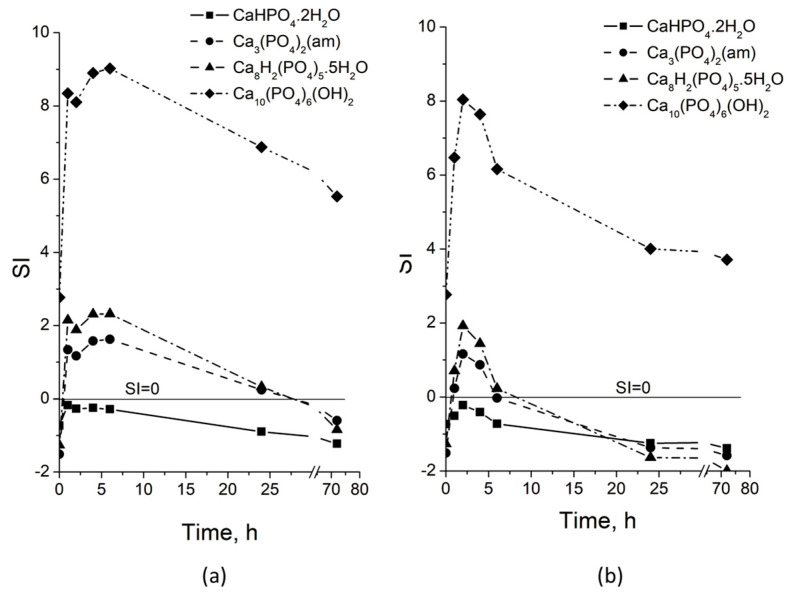
pHs and calculated saturated indices (SIs) of CaHPO_4_.2H_2_O, Ca_3_(PO_4_)_2_(am), Ca_8_H_2_(PO_4_)_6_.5H_2_O, and Ca_10_(PO_4_)_6_(OH)_2_ in the liquid phase during the maturation of CP-SBFgb (**a**) and CP-SBFgbGly (**b**).

The kinetic studies supported the thermodynamic calculation of saturation indices (SIs) of possible solid phases in the artificial saliva at different maturation times. The initial saliva (Figure 8) is only supersaturated (SI > 0) concerning the Ca_10_(PO_4_)_6_(OH)_2_. The dissolution of the amorphous phase leads to an increase in the saturated indices of Ca_3_(PO_4_)_2_(am), Ca_8_H_2_(PO_4_)_6_.5H_2_O, and Ca_10_(PO_4_)_6_(OH)_2_ to the 6th h for CP-SBFgbGly (Figure 8a) and to the 2nd h for CP-SBFgb (Figure 8b), making them positive, indicating supersaturation of the solutions concerning these salts. The subsequent decrease in SI values was related to spontaneous precipitation. The supersaturation concerning Ca_3_(PO_4_)_2_(am) and Ca_8_H_2_(PO_4_)_6_.5H_2_O is going after 6th h by CP-SBFgbGly and after 30th h by CP-SBFgb (SIs become negative). This reveals that higher concentrations of Mg and Zn in the CP-SBFgb (Table 3) stabilise the amorphous phase and delay its recrystallisation into the thermodynamically stable phase, hydroxyapatite, whose saturation index remained the highest and positive throughout the studied interval.

We hypothesise that the smaller effective ionic radii of Zn^2+^ (0.74 Å) and Mg^2+^ (0.71 Å) relative to Ca^2+^ (1.00 Å) [46] ions reduce the interatomic distances in the Posner clusters, making them more stable at contact with the artificial saliva solution.

## 5. Conclusions

A comparative study was performed on the precipitation of double-doped calcium phosphate in the modified simulated body fluid (SBF) and artificial saliva (AS). The obtained solid phases are promising components of systems for remineralising early carious lesions.

Thermodynamic modelling of chemical equilibria in the SBF/AS-NaCl-CaCl_2_-MgCl_2_-ZnCl_2_-K_2_HPO_4_-H_2_O and SBF/AS-NaCl-CaCl_2_-MgCl_2_-ZnCl_2_-K_2_HPO_4_-Gly/Val-H_2_O systems at pH 6.5, close to the AS, pH 7.5, close to the SBF, and 8.5 allow us to predict that the same calcium phosphates will precipitate in both biomimetic media, irrespective of electrolyte environment and the presence of Gly and Val. The latter affects the chemical composition of the precipitates.

The experimental study based on thermodynamic calculation shows only precipitation of X-ray amorphous cationic-deficient calcium phosphate at pH 8–8.2 independently of electrolyte medium, presence of organic molecules, and sequence of initial solution mixing. The constant pH during the syntheses and kinetic rather than thermodynamic factors are decisive for the precipitation of amorphous products. The concentration of Mg in the amorphous phase varies from 5.86 to 8.85 mol% and of Zn from 0.71 to 2.84 mol%, being the lowest in the sample obtained in the presence of Gly. The content of doped ions depends on the complexation in the solutions. The greater the number and more stable complexes formed in the solution, the lower the concentration of Zn and Mg in the solid phase.

Upon contact with artificial saliva, the amorphous product dissolves during the first 6 h, releasing ions, after which reverse deposition and recrystallisation begin. The higher concentration of Zn and Mg stabilises the amorphous phase and slows its transformation into hydroxyapatite. Thirty days are not enough for complete phase transformation into nanocrystalline hydroxyapatite.

Our research can serve as a basis for further in-depth in vitro studies of the remineralisation potential of double-doped with Zn and Mg calcium phosphates.

## Figures and Tables

**Table 1:** Composition and pH of the SBFc [3] and AS [26] and modified mSBFs and mASs, mmol·L^−1^.

Components	SBFc [3]	mSBFc1	mSBFc2	mSBFc3	AS [26]	mAS1	mAS2	mAS3
NaCl	136.8	128.4	141	141	9.92	9.92	9.92	9.92
KCl	3	3	3	3	17	506	17	17
CaCl_2_	2.5	0	378	2.5	1.5		378	
MgCl_2_	1.5	1.5	58	1.5			58	1.5
ZnCl_2_				13.2				13.2
Na_2_SO_4_	0.5	0.5	0.5	0.5				
NaHCO_3_	4.2	12.6						
K_2_HPO_4_	1	253			2.42	253		
Na_2_HPO_4_					3.03	3.03		
NH_4_Cl					2.99	2.99	2.99	2.99
NaSCN					1.98	1.98	1.98	1.98
Urea					3.33	-	-	-
Glucose					0.17	-	-	-
Ascorbic acid					0.01	-	-	-
Mucin, mg·L^−1^					2700	-	-	-
Gly *		2930	2930	2930		2930	2930	2930
Val *		512	512	512		512	512	512
pH	7.2–7.4	8.0–8.2	8.0–8.2	6.5	6.3–6.4	8.0–8.2	8.0–8.2	6.5

Note: * Gly and Val were added only in corresponding synthesis runs.

**Table 2:** Experimental conditions and product names.

Series	Precipitation Method	Precipitation Route	Products Name
A	Fast mixing	Simultaneously mixing of mSBFc1, mSBFc2, and mSBFc3	CaP-SBFf
		Simultaneously mixing of mSAS1, mAS2, and mAS3	CaP-ASf
B	Continuous precipitation (M (M = Ca, Mg, Zn)-containing solutions were added to phosphorous-containing solutions)	mSBFc2 and mSBFc3 added to mSBFc1	CaP-SBFcp
		mAS2 and mAS3 added to mSAS1	CaP-AScp
C	Continuous precipitation	Simultaneously addition of mSBFc2, mSBFc3, mSBFc1 to a glycine buffer [32]	CaP-SBFgb
		Simultaneously addition of mAS2, mAS3, mSAS1 to a glycine buffer [32]	CaP-ASgb
	Continuous precipitation in the presence of glycine	Simultaneously addition of mSBFc2, mSBFc3, mSBFc1 to a glycine buffer [32]	CaP-SBFgbGly
		Simultaneously addition of mAS2, mAS3, mAS1 to a glycine buffer [32]	CaP-ASgbGly
	Continuous precipitation in the presence of valine	Simultaneously addition of mSBFc2, mSBFc3, mSBFc1 to a glycine buffer [32]	CaP-SBFgbVal
		Simultaneously addition of mAS2, mAS3, mSAS1 to a glycine buffer [32]	CaP-ASgbVal

**Table 3:** Composition of the obtained calcium phosphates.

Samples	(Ca + Zn + Mg)/P	Mg/(Ca + Zn + Mg)	Zn/(Ca + Zn + Mg)
		mol%	
Series A			
CP-SBFf	1.60 ± 0.02	7.62± 2	2.54 ± 3
CP-ASf	1.40 ± 0.01	6.93 ± 2	2.98 ± 3
Series B			
CP-SBFcp	1.51 ± 0.02	8.54 ± 2	1.51 ± 2
CP-AScp	1.35 ± 0.01	8.85 ± 2	1.15 ± 2
Series C			
CP-SBFgb	1.57 ± 0.02	7.90 ± 2	2.84 ± 3
CP-ASgb	1.42 ± 0.02	7.64 ± 2	2.55 ± 3
Series C with Gly and Val			
CP-SBFgbGly	1.40 ± 0.01	5.86 ± 1	0.71 ± 2
CP-ASgbGly	1.40 ± 0.01	6.21 ± 1	0.82 ± 2
CP-SBFgbVal	1.54 ± 0.02	6.37 ± 1	1.73 ± 2
CP-ASgbVal	1.44 ± 0.01	7.25 ± 2	2.15 ± 2

## Data Availability

The data presented in this study are available in this article and Supplementary Materials.

## Supplementary Materials

The following supporting information can be downloaded at https://www.mdpi.com/article/10.3390/biomimetics9080455/s1, Figure S1: X-ray powder patterns of all synthesized samples and Figure S2: ^1^H spin-echo NMR spectra of CaP-SBFgbGly and CaP-SBFgb samples incubated at different time periods in SBF: parent samples (black), 1 h in SBF (red), 4 h in SBF (blue), 24 h in SBF (magenta), 30 days in SBF (green).
