# Supplementary material for: Thermodynamic and Kinetic Studies of the Precipitation of Double-Doped Amorphous Calcium Phosphate and Its Behaviour in Artificial Saliva
Source: Biomimetics (Basel). 2024 Jul 25;9(8):455. doi: 10.3390/biomimetics9080455 (PMC11351230; doi:10.3390/biomimetics9080455)
Supplement: Supplementary file 1 [file biomimetics-09-00455-s001.zip › biomimetics-3065869-supplementary.pdf]

Supplementary Material

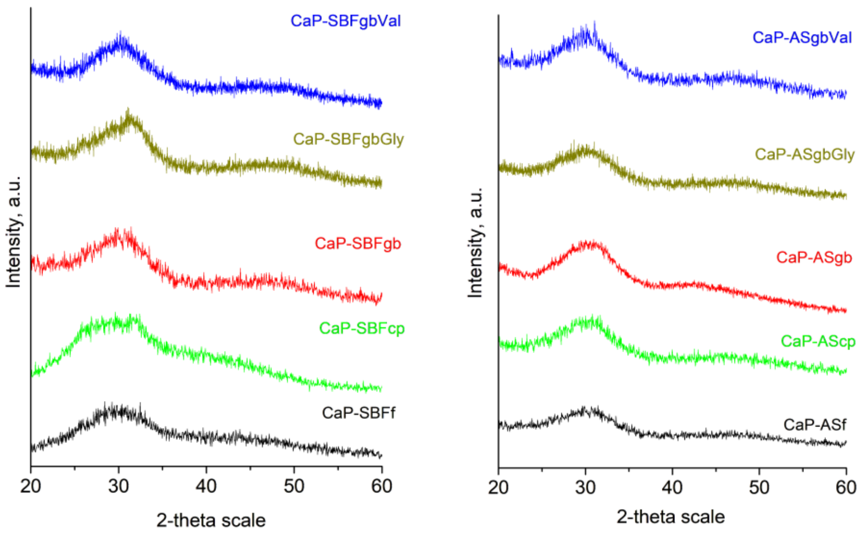

a b

Figure S1 X-ray powder patterns of all synthesized samples: (a) synthesized in SBF; (b) synthesized in AS

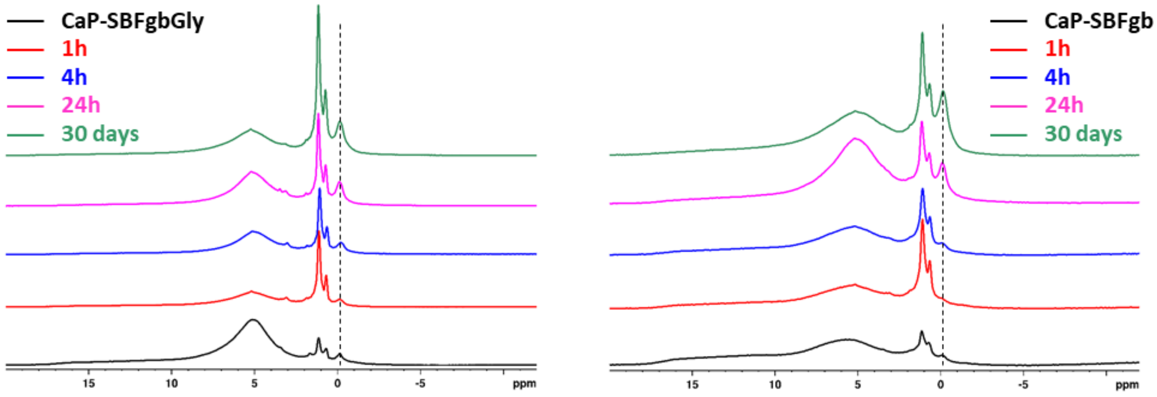

Figure S2: ^{1}H spin-echo NMR spectra of CaP-SBFgbGly and CaP-SBFgb samples incubated at different time periods in SBF: parent samples (black), 1h in SBF (red), 4h in SBF (blue), 24h in SBF (magenta), 30 days in SBF(green). The vertical dotted line indicates the increase of the characteristic resonance of the nanocrystalline HAp phase with the increase of the incubation time.
